# A Concise Review on the Molecular Structure and Function Relationship of β-Glucan

**DOI:** 10.3390/ijms20164032

**Published:** 2019-08-18

**Authors:** Bin Du, Maninder Meenu, Hongzhi Liu, Baojun Xu

**Affiliations:** 1 Hebei Key Laboratory of Natural Products Activity Components and Function, Hebei Normal University of Science and Technology, Qinhuangdao 066004, China; 2 Food Science and Technology Program, Beijing Normal University–Hong Kong Baptist University United International College, Zhuhai 519087, China; 3 Institute of Agro-products Processing Science and Technology, Chinese Academy of Agricultural Sciences, Beijing 100193, China

**Keywords:** β-glucan, molecular structure, functional properties, viscosity, solubility

## Abstract

β-glucan is a non-starch soluble polysaccharide widely present in yeast, mushrooms, bacteria, algae, barley, and oat. β-Glucan is regarded as a functional food ingredient due to its various health benefits. The high molecular weight (Mw) and high viscosity of β-glucan are responsible for its hypocholesterolemic and hypoglycemic properties. Thus, β-glucan is also used in the food industry for the production of functional food products. The inherent gel-forming property and high viscosity of β-glucan lead to the production of low-fat foods with improved textural properties. Various studies have reported the relationship between the molecular structure of β-glucan and its functionality. The structural characteristics of β-glucan, including specific glycosidic linkages, monosaccharide compositions, Mw, and chain conformation, were reported to affect its physiochemical and biological properties. Researchers have also reported some chemical, physical, and enzymatic treatments can successfully alter the molecular structure and functionalities of β-glucan. This review article attempts to review the available literature on the relationship of the molecular structure of β-glucan with its functionalities, and future perspectives in this area.

## 1. Introduction

**Figure 1:**
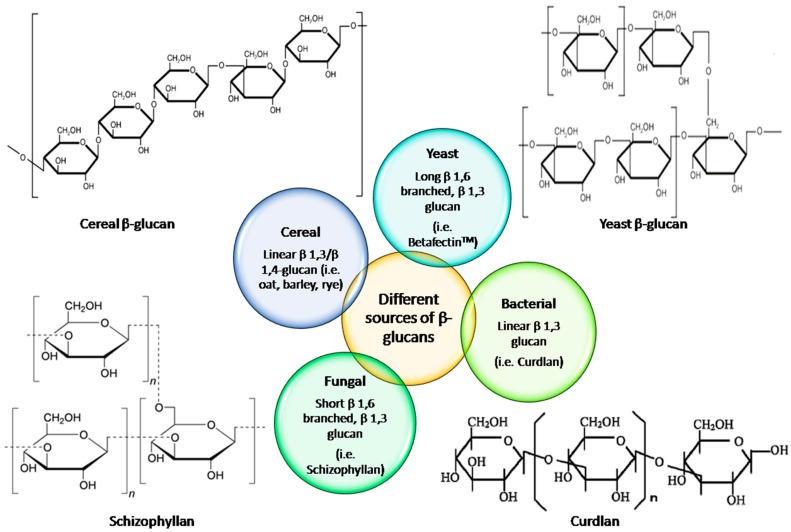
Structure and branching degree of β-glucan from different sources.

β-glucans are polysaccharides of d-glucose monomers linked through β-glycosidic bonds, and are widely present in yeast, fungi (including mushrooms), some bacteria, seaweeds, and cereals (oat and barley) [1,2]. β-Glucan is acknowledged as a functional and bioactive food ingredient owing to its biological activities, such as hypocholesterolemic, hypoglycemic immunomodulatory, antitumor, antioxidant, and anti-inflammatory activities [3,4]. Primarily, β-glucan is a linear polymer composed of d-glucopyranosyl units connected by isolated β-(1 → 3) linkages or a set of β-(1 → 4) linkages. The β-(1 → 3) linkages confer a wormlike coil conformation to the *β*-glucan [5]. β-Glucans exhibit significant differences in their macromolecular structure based on their sources. The structure and description of β-glucan from various sources are presented in Figure 1 [6].

Lentinan, a β-glucan from fungus *Lentinus edodes*, exists as triple helical structures at room temperature, resulting in its high viscosity and excellent tolerance to a broad range of pH, temperature, and salt concentrations in aqueous solution [7]. Ohno et al. [8] reported that high molecular weight β-glucan (schizophyllan) exists in single or triple helix conformations. In contrast, the low molecular weight β-glucan exhibits a random coiled conformation. The molecular and structural characteristics of β-glucan have attracted the strong interest of researchers because they determine its physical properties, such as water solubility and rheological behavior, as well as the functional effects on food products [9,10,11]. Lei et al. [12] reported that yeast β-glucan with low molecular weight had better antioxidant and immunological activities. However, Suárez et al. [13] found that the higher molecular weight β-glucan from *Chlorella pyrenoidosa* exhibited immunostimulatory activity.

β-Glucan is also extensively used in the food industry for its ability to form a gel and enhance the viscosity of aqueous solutions. It is used for enhancing the texture and appearance of salad dressings, gravies and ice creams. β-Glucan is also used as a fat mimetic to develop calorie-reduced food products [14]. However, the flow behavior and gelling properties of β-glucan cause several technical problems for food industries, such as slow filtration of solutions or slurries, low yield, and precipitation during beer storage. Thus, the applications of β-glucan in the food, cosmetics, and pharmaceuticals industries have been limited due to its high molecular weight and viscosity [15,16]. Various studies have reported that the application of chemical, enzymatic, and physical methods for altering the molecular structure of β-glucan imposes a significant impact on its solubility, viscosity, and other rheological parameters [17,18,19,20,21].

In recent years, our group has published various review papers related to the effect of β-glucan on skin health and the anti-inflammatory effects of β-glucan, and has also published a review paper on the production and industrial applications of β-glucan [2,22,23]. However, no review paper is available on the relationship between molecular structure and the functionality of β-glucan. Nevertheless, a concise understanding and analysis of available literature regarding the relationship between the molecular structure and functionality of β-glucan are crucial for its successful application in the food and pharmaceutical industries. In the following sections, a concise relationship between molecular structure and functionality of β-glucan has been presented.

## 2. The Molecular Structure of β-Glucan

### 2.1. Molecular Weight

The molecular weight (Mw) of β-glucan was reported to be in the range of 21–1100 × 10^3^, 31–2700 × 10^3^, 65–3100 × 10^3^, 209–487 × 10^3^ g/mol in the case of rye, barley, oat, and wheat, respectively [24]. Table 1 and the following sections summarize the different methods employed for the determination of Mw of β-glucan and variation in its Mw based on their sources.

Currently, size exclusion chromatography (SEC) is regarded as the most convenient method for the determination of the molecular weight of lentinan, its distribution, and conformational parameters without standard samples by employing on-line detection of concentration using refractometer and multi-angle laser light scattering (SEC–MALLS) [17]. Previously, researchers have also attempted to determine the molecular weight and its distribution in Schizophyllan by employing SEC coupled with a refractive index (RI) detector and the MALLS method [16]. The average Mw of polysaccharides from *Pavlova viridis* was determined by employing SEC with two types of columns in series. The standard curve of Dextran T of known Mw was employed to determine Mw of polysaccharides [25]. The molecular size and distribution of β-glucan from oat and cereals, (1 → 3, 1 → 4)-β-glucan from barley, hydrolyzed β-glucan extracts, and β-glucan from muffins were determined by employing a high-performance size exclusion chromatography (HPSEC) [26,27,28,29,30,31]. SEC was also used for determination of Mw of the partially hydrolyzed oat β-glucan, sulfated-derivatized oat β-glucan, and β-d-glucan extracted from baker’s yeast [19,20,32]. Another study also employed SEC–MALLS for determination of true Mw and distribution of β-d-glucan from *Lentinus edodes* [33]. SEC, along with laser light scattering, was also employed to elucidate Mw of β-glucan isolated from *Auricularia auricula-judae* [34]. Ulmius et al. [10] used asymmetrical flow field-flow fractionation (AsFIFFF) for the characterization of bacterial β-glucan. The molar mass distribution was determined by connecting the AsFIFFF in-line to a multi-angle light scattering (MALS) detector coupled with an RI detector. Zheng et al. [35] employed static light scattering and dynamic light scattering to characterize the Mw of triple-helical β-glucan isolated from *L. edodes* by using a Zimm plot. Moreover, researchers have also attempted to determine the Mw of barley β-glucan based on the intrinsic viscosity measurements [36].

The large variation in the Mw of β-glucan is due to the diversity in its source, extraction protocol, and methodology employed for its determination. It was also mentioned that the Mw of β-glucan is also dependent on the solvents, reaction conditions, sample history, detectors, and standard compounds [24,37].

### 2.2. Conformation

The inter-molecular and intra-molecular forces such as hydrogen bonds execute a decisive role in the conformation of polysaccharides. Polysaccharides are known to exist in various conformations such as single helix, double helix, triple helix, random coil, aggregate, rod-like shape, and worm-like shape [50]. The conformational characterization of β-glucan was done based on its Mw, radius of gyration, and intrinsic viscosity calculated from SEC-LLS and viscometry measurements. These values were further used to calculate the molar mass per unit contour length (ML) and persistence length (q). The values for ML and q present the conformation of β-glucan [33,38,39,40]. Previously, ML and q values of sulfated β-glucan collected from sclerotia of *Pleurotus tuber*-regium were reported to be 990 nm^−1^ and 8.5 nm, respectively. The higher value for q presented an expanded flexible chain of β-glucan in phosphate buffer saline [38]. Based on current theories for a wormlike chain model, the ML and q values of the β-glucan were calculated to be 633 nm^−1^ and 5.5 nm, respectively. The characteristic ratio was mentioned as being 20.2. These results indicated that water-insoluble (1 → 3)-β-d-glucan exists as a relatively extended flexible chain in 0.2 M NaCl [39]. In a recent study, the chain conformation of β-glucan from *Ganoderma lucidum* was measured. The ML, q, and contour length (h) per main-chain glucose residue were reported as 2150 nm^−1^, 128 nm, and 0.30 nm, respectively, indicating the triple-helical chains of glucan in PBS [40].

Some recent studies have also reported the degradation of β-glucan into small fragments exhibiting a wide range of molecular weights while retaining the native chemical structure and conformation [33,41]. Chemical methods, enzymatic methods, and physical methods were the different degradation methods employed for attaining β-glucan with lower Mw. Chemical methods involved the use of hydrogen peroxide treatment for oxidative degradation as well as acid or alkali-induced degradation [51]. Enzymatic methods involve treatment with cellulose, lichenase, or lichenase combined with amylase and/or proteinase [32,52]. Thermal and mechanical methods exhibit an advantage over the other methods as there is no need to add additional substances to the polymer. Thus, this eliminates the need for subsequent purification processes. Whereas, the chemical and thermal methods led to the generation of unwanted mono and oligomers [51]. However, the physical methods such as ultrasonic treatment were reported to lyse the polymer chain from the middle without leading to any side reactions. Thus, the physical methods have attracted huge attention from researchers for degradation of β-glucan into small fragments [23,51].

### 2.3. Branching Degree

The distribution of cellulosic oligomers in β-glucan was determined by lichenase treatment and chromatographic methods. The small oligosaccharides segment of β-glucan released by lichenase action was analyzed by high-performance anion-exchange chromatography (HPAEC) combined with pulsed amperometric detection [27,28,43,44,47,53]. In contrast, the chemical structure of chemically derivatized *β*-glucan was also determined by Fourier transform infrared and Raman spectroscopy [38,43].

β-Glucan is a polysaccharide comprised of linear chains of glucose with β-(1 → 3) and β-(1 → 4) linkages in endosperm cell walls of cereal grains. In contrast, the β-glucan present in yeast and fungi was composed of (1 → 3) linkages and (1 → 6) linked branches. The functionality of β-glucan basically depends upon types of linkages, degree of branching, and structural arrangement that, in turn, manipulate its biological activity [54]. It is mentioned that the introduction of suitable ionic groups with appropriate degrees of substitution enhanced the water solubility of the polysaccharides along with the change in the conformation of the polymer chain in a solution that, in turn, was responsible for their improved biological activities [42]. Previously, the degree of substitution (DS) of sulfated derivatives of β-glucan from sclerotia of *Pleurotus tuber-regium* was measured using elemental analysis and observed to be 1.14–1.74 [38]. The DS of carboxymethylated glucan determined by nuclear magnetic resonance (NMR) spectroscopy and reported to be 1.27 [39]. Table 2 presents the branching degree and conformation of β-glucan from different sources.

### 2.4. Monosaccharide Composition

The monosaccharide composition of polysaccharides was analyzed by employing gas chromatography equipped with a flame ionization detector [41]. The presence of monosaccharides other than glucose further increased the complexity of glucan (heteroglucans) [59]. Du et al. [41] determined the monosaccharide compositions of β-glucan from the submerged mycelial culture of *Schizophyllum commune*. The exopolysaccharide was reported to be a heteropolysaccharide containing galactose, mannose, xylose, arabinose, rhamnose, ribose, and glucose as the main monosaccharide (57.5%).

## 3. Functionalities of β-Glucan

The molecular and structural characteristics of β-glucan are the crucial parameters that determine physical properties and functional effects of β-glucan on food products [9].

### 3.1. Physicochemical Properties of β-Glucan

#### 3.1.1. Bile Acid-Binding Capacity

Bile acid is produced in the liver from the cholesterol. The binding of β-glucan with bile acid and its fecal excretion lead to decrease in the cholesterol levels in the body [60]. Kim and White [29] evaluated the impact of β-glucan Mw on in vitro bile-acid binding. The results showed that the low-Mw β-glucan (1.56 × 10^5^ g/mol) extracted from oats bound more bile acid compared to the high-Mw β-glucan (6.87 × 10^5^ g/mol). Another study reported that the β-glucan with Mw ranging from 2.42 × 10^5^ to 1.61 × 10^5^ g/mol bound maximum amount of bile acid. However, in this study, the β-glucan with highest Mw and lowest Mw bound the lowest amount of bile acid [30]. It was also mentioned that the muffins with high-Mw β-glucan (2.39 × 10^5^ g/mol) bound more bile acid compared with the muffins with low- (0.62 × 10^5^ g/mol) and medium-Mw β-glucan (1.20 × 10^5^ g/mol) [31]. Another study also studied the bile acid-binding capacity of β-glucan hydrolyzed by cellulase. It was mentioned that the β-glucan with Mw of 370,000 and 730,000 g/mol presented the highest values for bile acid-binding capacity (26.54% and 25.47%). However, β-glucans with obvious variation in their Mw did not exhibit an obvious variation in its bile acid-binding capacity [32]. Thus, it can be concluded that the bile acid-binding capacity of β-glucan is dependent on its Mw as well as the structural and physicochemical properties.

Recently, the chemical modification of β-glucan such as sulfation and acetylation has also gained significant attention. This may lead to the alteration in the basic structure and inter-molecular forces of β-glucan and result in the conformational transformation and change in the functional properties of β-glucan. It was reported that the sulfation of β-glucan reduced the Mw of oats β-glucan, that in turn, exhibit reduced bile acid-binding capacity [20]. Whereas, acetylation of oats β-glucan resulted in compact hole-less microstructure and enhancement in its bile acid-binding capacity [21]. Recently, it was also reported that gamma irradiation also leads to a significant reduction in the average Mw of yeast β-d-glucan with an increase in the irradiation dose that eventually enhances its bile acid-binding capacity [19].

#### 3.1.2. Solubility of β-Glucan

Polysaccharides have a strong affinity towards water molecules due to the presence of multiple-OH groups. The solubility of the β-glucan is an important parameter as it plays a crucial role in their functional activities, such as stability, emulsifying property, drug delivery, and membrane-forming properties [61]. A previous study reported that oat β-glucan with Mw ranging from 2.42 × 10^5^ to 1.61 × 10^5^ g/mol exhibit highest water solubility from 83.4% to 87.3% [30]. Another study reported that an increase in Mw of oat β-glucan from 1.13 × 10^5^ to 9.04 × 10^5^ g/mol and from 1.65 × 10^5^ to 8.51 × 10^5^ g/mol leads to a significant decrease in its water solubility from 72.8% to 68.2% and from 72.3% to 67.2%, respectively [55]. The chemical modification of β-glucan, namely sulfation, incorporates ionic groups and also enhances the amount of small fragment of β-glucan that, in turn, lead to a decrease in Mw from 130,000 to 68,000 g/mol and an increase in its solubility from 20.3% to 42.3% [20].

Gamma treatment of β-glucan causes radiolysis of the glycosidic bond and reduction in the Mw of β-glucan from 178,000 g/mol in the case of non-irradiated sample to 62,000, 32,000 and 25,000 g/mol in case of sample irradiated at 10, 30, and 50 kDa that, in turn, lead to an improvement in its solubility from 51.30% to 55.76%, 75.81%, and 81.72%, respectively [18]. Ultrasonication was also reported as another physical method for producing a homologous series of lower Mw polysaccharides [23,62]. Ultrasonic treatment of β-glucan resulted in polymer degradation and a significant increase in its solubility [62]. This increase in the solubility of β-glucan would be of great interest to the food and pharmaceutical industries. However, previous researchers have also reported the high number of cellotriosyl oligomer blocks as a reason behind the low solubility of barley β-glucan [43].

### 3.2. Rheological Properties of β-Glucan

The rheological analysis was observed to a significant method for understanding the structure-function relationship of polysaccharides in aqueous media. It was mentioned that even though the chemical structure of some polysaccharides, such as κ-carrageenan, ι-carrageenan, and agarose, are similar to each other, their rheological and gelling properties are quite different. The shear-thinning behavior was dependent on the Mw and concentration of the β-glucan preparations [63]. Previously, it was indicated that the β-glucan samples with low molecular weights displayed the gel-like properties attributed to the self-association via cellulose-like sequences [53]. The purified water-soluble barley (1 → 3, 1 → 4)-β-glucan with higher Mw presented higher yield stress and lower compression modulus values. In contrast, the dynamic storage modulus value was decreased with increase in the molecular size [28].

The large deformation mechanical tests up to failure revealed that an increase in the molar ratios of tri to tetrasaccharides and molecular size of β-glucan reduced the brittleness and increased the strength of mixed-linkage β-glucan gels [44]. The large deformation mechanical tests (compression mode) showed that an increase in molecular size and decrease in trisaccharide units was associated with an increase in the strength of β-glucan cryogels [26]. It was also reported that freshly prepared low-Mw and high-Mw oat β-glucan solutions presented similar random coil type rheological characteristics. An increase in the rate of gel structure development was also mentioned with a decrease in the molecular size of the β-glucan [27]. Previously, researchers have also confirmed the variation in the Mw of water-extractable *β*-glucan from various cultivars of barley based on the differences in their viscoelastic behavior [45].

#### 3.2.1. Gel Property

The studies on the β-glucan showed that the β-glucan sample with different Mw exhibit variation in their gelation rate. Skendi et al. [46] extracted β-glucan from milled seeds of two oat cultivars. The dynamic rheometry revealed that all of the β-glucan samples under investigation were able to form gels. The oat β-glucan with low Mw presented high gelation rates and short gelation times compared to its high-Mw counterparts. Earlier, it was also mentioned that β-d-glucan with lower Mw forms a gel more easily compared to the β-d-glucan with high Mw [37]. The high mobility and high structural regularities of low-Mw β-glucan lead to an easy formation of junction zones and stronger three-dimensional network that, in turn, enhances its gelation rate [56]. Another study on wheat β-d-glucan reported an increase in the gelation rate with a decrease in the Mw of β-glucan due to less spatial hindrance and higher mobility of shorter chains [57]. The gelation time of the purified water-soluble barley (1 → 3, 1 → 4)-β-glucan exhibiting low molecular size was reported to be shorter than the β-glucan with high molecular size [28].

In another study, Brummer et al. [47] evaluated gel properties varied with the proportion of high-Mw (1,190,500 g/mol) and low-Mw (31,200 g/mol) *β*-glucan extracted from oat. The mix of 50% high Mw and 50% low Mw produced the hardest, but also the most elastic, gels. The compact hole-less microstructure of oat β-glucan in response to acetylation resulted into lack of adhesiveness, lower hardness, increased cohesiveness, springiness and gumminess [21]. These characteristics of β-glucan improve its application at industrial level and fiber’s dispersion and solubilization in food applications. It was also mentioned that decrease in the Mw from 213 × 10^−3^ to 0.10 × 10^−3^ g/mol of mixed-linkage (1 → 3) (1 → 4)-β-d-glucan from oat and barley resulted in the decreased gelation time and increase in the gelation rate [44].

Partial hydrolysates of (1 → 3)(1 → 4)-β-d-glucan from oats with cellulose cleaved the molecule with longer contiguous β-(1 → 4)-linked d-glucopyranosyl units and resulted in a decrease in its Mw from 1,190,500 to 30,800 g/mol. That, in turn, resulted in more elastic gels with stronger junction zones compared to the partial hydrolysates of molecules produced with lichenase that targets the β-(1 → 4) glycosidic 3-o-substituted glucose links [48]. Aggregation of β-glucan in aqueous solutions was mentioned to be diffusion limited. It was reported that an increase in the Mw from 103,500 to 2,076,000 g/mol of cereal β-glucan leads to a reduced degree of aggregation attributed to the inferior diffusion rate of large molecules. β-Glucan with more rigid conformation (higher tri/tetra ratio) also exhibit lower degrees of aggregation [57].

#### 3.2.2. Viscosity

**Figure 2:**
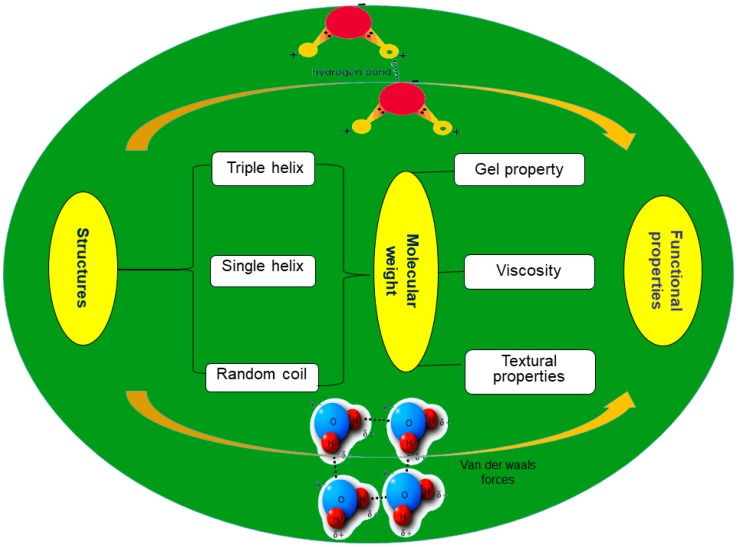
The relationship between structure and function of β-glucan.

β-Glucan contributes to the viscosity of the solution [29]. The intrinsic viscosity of a solution is also regarded as a characteristic property of the polysaccharide solution. The viscosity, in turn, suggested to be dependent on the molar mass of the polysaccharides, the concentration, and the ability to form aggregates [64]. Figure 2 presents the relationship between molecular structure and functionality of β-glucan. It was mentioned that β-glucan increases the viscosity of the gastrointestinal tract and delay the gastric emptying as well as the intestinal absorption of carbohydrates, that in turn, led to reduced post-prandial hyperglycemia as well as the absorption and reabsorption of cholesterol and bile acids [24]. Various studies have presented the high correlations between Mw of β-glucan and the viscosity of solutions. Kim and White [55] have also reported an increase in the viscosity with an increase in the Mw of oat β-glucan. It was also reported that the gamma irradiation of β-glucan lead to radiolysis of glycosidic bonds that lead to a decrease in its Mw and ultimately reduces the viscosity of β-glucan [18]. In another study, the decrease in the Mw of gamma-irradiated yeast β-d-glucan was also reported to be responsible for the decrease in its viscosity [19]. In contrast, acetylation of oat β-glucan was mentioned to form compact pore-less microstructure and resulted in the reduced viscosity [21].

Another study reported the reduced Mw, a drastic decrease in the viscosity and an increase in the solubility of β-glucan following ultrasonic treatment [58,62]. Thus, gamma irradiation, ultrasonic treatment, and acetylation of β-glucan can be employed as an effective method to address the physical problems involved in the use of β-glucan in food and pharmaceutical industry due to its high viscosity and low solubility. The high-Mw β-glucan was reported to stabilize the emulsions by enhancing the viscosity of the continuous phase. However, the stability of emulsion due to the low-Mw β-glucan was mainly attributed to the network formation in the continuous phase [65]. It was also mentioned that the steaming of oat grain leads to changes in the conformation of native β-glucan by disrupting intramolecular cross-linking to a linear chain configuration that, in turn, generate higher viscosity and highly pseudoplastic behavior [63]. Another study on oat β-glucan also revealed that samples with higher Mw exhibit higher viscosity at the same concentration [66].

#### 3.2.3. Textural Properties

Fortification of cereal and dairy-based foods with β-glucan has illustrated the potential of β-glucan to manipulate food structure, texture, and acceptability. Researchers have also explored the impact of β-glucan molecular structure on the texture and overall acceptability of food products. Sayar et al. [67] added β-glucan extracts to plain muffin formulations to investigate the effect of Mw of β-glucan on textural properties of the muffins. The results showed that the addition of β-glucan at the maximum limit for equivalent batter firmness, the muffins in the low-Mw (57,000 g/mol) treatment group was firmer and less springy than the high-Mw (560,000 g/mol) group. It was also mentioned that the addition of hydrolyzed oat β-glucan with 10% fat-rich meatball present higher overall acceptability compared to the meatballs with native oat β-glucan [49]. Table 3 summarizes the functional properties of β-glucan collected from various sources.

## 4. Industrial Application of β-Glucan

β-Glucan has been extensively used in food and pharmaceutical industries due to its physical properties such as water solubility, viscosity, and gelation [78]. Previously, high-Mw oat β-glucan was incorporated into milk, resulting in calorie-reduced and cholesterol-lowering dairy products [79]. Researchers have also observed that the yoghurt with β-glucan exhibited a high proportion of free amino acids, faster proteolysis, and lower release of large peptides compared to the yoghurt with starch and without β-glucan [80]. Prebiotic sausages have also been formulated by a combination of β-glucan, resistant starch, and starch [81]. The ready-to-eat snacks with β-glucan fractions from barley and mushroom have also been formulated and an improved glycemic response was also reported after consuming these snacks [82]. A sucrose-sweetened beverage with 3 g barley β-glucans was also reported to control food intake and reduce 24 h energy intake [83]. Researchers have also prepared β-glucan-enriched materials, a wheat flour substitute, from low-quality mushroom *Lentinus edodes.* The cakes with 1 g of β-glucan-enriched materials per serving resulted in the quality attribute similar to that of the control sample [84]. β-Glucan was also incorporated into rusks and bread prepared by rice starch and barley. That results in the formulation of functional food products and snacks. These functional products are cherished by consumers for their hypocholesterolemic and hypoglycemic properties [85,86].

Recently, cyclic glucan and composite of β-glucan and chitosan have also been used as wound dressing material [87,88]. β-Glucan was also used as an effective therapeutic agent for the treatment of burn injuries [89,90]. Another study has reported that the application of poly(lactic-co-glycolic acid) membranes containing β-glucan result into the accelerated wound healing and these membranes were also reported to be used as a skin substitute [91]. In contrast, the flexible hydroxyapatite/glucan composite was reported to have the potential to be used as bone-substituting material [92]. The carboxymethylated β-glucan also reported to be employed in arthritis treatment [93]. Another study by Anusuya and Sathiyabama [94] also reported the antifungal potential of nanoparticles prepared from β-glucan isolated from the cell wall of *Pythium aphanidermatum*. Researchers have also suggested the use of β-glucans collected from cap-opened, more fragile mature fruiting bodies of *Agaricus brasiliensis* for the production of high-quality nutraceuticals [95].

## 5. Conclusions and Future Perspectives

β-Glucan, a non-starch polysaccharide, is a principal functional ingredient widely present in barley and oats. The available literature revealed that the functionality of β-glucan is chiefly dependent on its Mw, conformation, linkage-type, 1 → 3, 1 → 6 linkage ratio, length, number, and chemical or physical modification. Along with these parameters, the amount and nature of co-extracted compounds in a β-glucan preparation also influences its solubility, aggregation, and conformation significantly that eventually alter the functionality of β-glucan [96]. The triple helix of polysaccharide was dissociated to the single helix in response to physical and chemical treatments due to the breakage of inter and intramolecular hydrogen bonds that, in turn, lead to a reduction in its Mw, increase in solubility, and decrease in the viscosity. This enhancement in the solubility and reduction in viscosity β-glucan is of immense importance in the food and pharmaceutical industries where high viscosity and low solubility of β-glucan hinder its application in final product formulation. A plethora of research work has reported that the Mw of a β-glucan exhibits a great influence on its physicochemical properties, that, in turn, affect its biological activities. Fortification of food products with low-Mw β-glucan enhances the overall acceptability of food product. However, the exact and detailed mechanism behind the impact of β-glucan structure on its function has yet to be explored. Nevertheless, it can be concluded that chemical and physical treatment of β-glucan and certain extraction procedures resulted in lower-Mw β-glucan that can be useful for the food industries for the production of novel functional food products.

## Figures and Tables

**Table 1:** Molecular weight of β-glucan extracted from various sources.

Source of β-Glucan	Determination Methods	Chromatographic Conditions		Standard Used	Molecular Weight (g/mol)	References
		Mobile Phase	Column			
*Schizophyllum commune* Fr. ACCC51174	HPLC-MALLS-RI	0.1 M NaNO_3_ at 0.5 mL/min	OCpak SD-822 M ZQ	--	808,000–240,4000 Pd: 1.18–1.86	[16]
Lentinan	SEC–MALLS-DRI	0.15 M aq NaCl at 0.50 mL/min	TSK-GEL G4000 PWXL and G6000 PWXL at 25 °C	--	14.6 × 10^−4^–163.5 × 10^−4^	[17]
*Saccharomyces cereviseae*	SEC-RI	--	--	Dextrans	279,00–175,000	[19]
Oat	SEC-RI	Distilled water at 3.5 mL/min	JAIGEL-W254, JAIGEL-W-253, JAIGELW252	--	68,000–130,000	[20]
*Schizophyllum commune*	HPLC-RI	0.1 M NaNO_3_ at 0.8 mL/min	PL aquageloh MIXED-H	--	197,000–290,0000	[23]
Oat, wheat, barley	HPSEC-RI	0.15 M NaNO_3_ containing 0.02% NaN_3_ at 0.5 mL/min	TSK G5000 PW-SEC at 25 °C	β-glucan standards from Megazyme	65 × 10^3^–200 × 10^3^	[26]
Oat	HPSEC-RI	0.15 M NaNO_3_ containing 0.02% NaN_3_ at 0.5 mL/min	TSK G5000 PW-SEC at 25 °C	β-glucan from Megazyme	35 × 10^−3^–250 × 10^−3^	[27]
Barley	HPSEC-RI	0.15 M NaNO_3_, containing 0.02% NaN_3_ at 0.5 mL/min	TSK G5000 PW-SEC at 25 °C	(1 → 3, 1 → 4)-β-glucan from Megazyme	40 × 10^3^–250 × 10^3^	[28]
Oat	SE-HPLC-RI	Milli-Q water with 0.02% sodium azide at 0.5 mL/min	Ohpak SB-806 HQ, Ohpak SB-805 HQ and Ohpak SB-804 HQ at 40 °C	β-glucan from Megazyme	1.56 × 10^5^–6.87 × 10^5^	[29,30]
Oat	SEC	Deionized water at 3.5 mL/min	JAIGEL-W254–255 at 25 °C	Dextran	370 × 10^3^-1450 × 10^3^	[32]
*Lentinus edodes*	SEC-MALLS-DRI	0.9% aqueous NaCl and Me_2_SO at 1.00 mL/min	TSK-GEL G6000 PWXL, G4000 PWXL, G4000-H8, G3000H8 at 25 °C	No standard sample was employed	1.87 × 10^−5^–28.3 × 10^−5^	[33]
*Pleurotus tuber-regium*	SEC-LLS and interferometric refractometer	PBS at 1.0 mL/min	PSW5000 and PSW3000 at 37 °C	--	5.76 × 10^4^–77.4 × 10^4^ Pd: 1.55–1.83	[38]
*Poria cocos*	SEC-LLS-DRI	0.2 M NaCl at 1.0 mL/min	TSK-GEL G5000 and G3000 PWXL at 25 °C	--	6.1 × 10^−^^4–^45.4 × 10^−^^4^ Pd: 1.3–1.7	[39]
*Ganoderma lucidum*	HPSEC-MALLS-RI-VS	0.15 M NaNO_3_, 0.05 M NaH_2_PO_4_, and 0.02% NaN3 at 0.5 mL/min	TSK G6000 PWxl, TSK G4000 PWxl at 30 °C	--	24.2 × 10^5^–2.9 × 10^5^ Pd: 1.22–1.83	[40]
*Schizophyllum commune*	HPLC-RI	0.05mol/L phosphate buffer (pH 6.7) containing 0.05% NaN_3_ at 0.5 mL/min	TOSOHTSK-GEL G3000 SW XL at 35 °C	Dextran	2,900,000	[41]
*Ganoderma lucidum*	SEC-LLS-RI	0.2 M NaCl at 1.0 mL/min	TSK-GEL G4000 PWXL at 25 °C	--	5.7 × 10^−^^4^–44.5 × 10^−^^4^ Pd: 1.8–2.2	[42]
Barley and oat	HPSEC-RI	0.05 M NaCl at 0.5 mL/min	Ultrahydrogel 1000 and 2000 at 60 °C	β-glucan from Megazyme	130,000–390,000 and 190,000–410,000	[43]
Oat and barley	HPSEC-RI	0.15 M NaNO_3_ containing 0.02% NaN_3_ at 0.5 mL/min	TSK G5000 PW-SEC at 25 °C	β-glucan from Megazyme	105 × 10^−^^3^–213 × 10^−^^3^	[44]
Barley	HPSEC-MALLS-RI-UV detector	0.15 M NaNO_3_ containing 0.02% NaN_3_	TSK G5000 PW at 25 °C	--	0.22 × 10^−^^6^–2.45 × 10^−6^ Pd: 2.0–7.0	[45]
Oat	HPSEC-MALLS-RI	0.15 M NaNO_3_ containing 0.02% NaN_3_ at 0.4 mL/min	TSK G5000 PW at 25 °C	Pullulan	0.18 × 10^−6^–0.85 × 10^−6^ Pd: 1.50–2.39.	[46]
Wheat	HPSEC-RALLS-DV-RI	0.1 M NaNO_3_ containing 0.03% (*w*/*w*) NaN_3_ at 0.6 mL/min	Shodex Ohpak KB-806M and Ultrahydrogel linear at 40 °C	--	0.43 × 10^5^–7.58 × 10^5^ Pd: 1.03–1.26	[37]
Oat	HPSEC-RI-DP-LLS	0.1 M NaNO_3_ with 5 mM NaN_3_ at 0.6 mL/min	Ultrahydrogel linear column, and Shodex OHpak Kb-806M at 40 °C	Pullulan	31,200–1,190,500 Pd: 1.20–1.27	[47]
Oat	SEC with Viscotek triple detector	100 mM NaNO_3_ containing 5 mM NaN_3_ at 0.6 mL/min	Shodex Ohpak Kb-806M at 40 °C	Pullulan	30,800–1,190,500 Pd: 1.20–1.73	[48]
Oat	HPSEC system with refractive index detector	Ultrapure water with 5 mM NaN_3_ at 0.8 mL/min	OHpak SB-804HQ at 30 °C	Dextran	0.06 × 10^3^–9.4 × 10^8^ Pd: 1.1–11.4	[49]

HPLC, high performance liquid chromatography; MALLS, multiangle laser light scattering method; RI, refractive index detector; SEC, size-exclusion chromatography; DRI, differential refractive index; HPSEC, high-performance size exclusion chromatography; SE-HPLC, size-exclusion high-performance liquid chromatography; LLS, laser light scattering; Pd, polydispersity index; VS, viscosity detector; RALLS, right-angle laser light scattering detector; DV, differential viscometer; DP, differential pressure.

**Table 2:** Conformation and branching degree of β-glucan from different sources.

Source	Conformation	Branching Degree	References
Barley	--	Linear chains of β-d-glucopyranosyl units linked via (1 → 3) and (1 → 4) linkages.	[10,28,43]
Schizophyllan from *S. commune* Fr. ACCC51174	--	Linear chain of β-d-(1 → 3)-glucopyranosyl groups and β-d-(1 → 6)-glucopyranosyl groups	[16]
Lentinan from *Lentinus edodes*	Triple helix in 0.15 M aq NaCl sulfated derivative exists as single semi-stiff chains in 0.15 M aq NaCl	β-(1 → 3)-d-glucan bearing β-(1 → 6)-d-glucopyranosyl branches	[17,33,35]
*β*-glucan from *Saccharomyces cereviseae*	--	Linearly linked β-d-glucopyranosyl units with (1 → 3) and (1 → 6) linkages	[19]
Oat	--	Unbranched polymers composed of (1 → 3)-and (1 → 4)-β-d-glucose units with (1 → 4) *β*-linkage predominating.	[20,27,29,30,43,47,53,55]
Oat, barley, and wheat	Rigid, rod-like conformation	Mixed-linkage linear (1 → 3), (1 → 4)-β-d-glucan	[26,44,56,57]
*β*-glucan from *Auricularia auricular-judae*	Semi-stiff conformation	(1 → 4)-linked d-glucopyranosyl with branching points at O-6 of (1 → 6)-linked d-glucopyranosyl residues	[34]
*β*-glucan from *Pleurotus tuber-regium*	Expanded flexible chain in PBS	Main chain of (1 → 3)-β-d-glucopyranosyl units with every third unit having on average a (1 → 6)-β-d-glucopyranosyl branch.	[38]
*β*-glucan from *Poria cocos*	Extended flexible chain in 0.2 M NaCl	(1 → 3)-β-d-glucan	[39]
*β*-glucan from *Ganoderma lucidum*	Triple-helical conformation with high rigidity	β-(1 → 3)-d-glucan with β-(1 → 6) branches	[40,42]
Chitosan	--	(1 → 4)-2-amino-2-deoxy-β-d-glucan	[58]
Oat	More extended and stiffer conformation for the low-Mw *β*-glucans	Unbranched polymers composed of (1 → 3)-and (1 → 4)-β-d-glucose units with (1 → 4) β-linkage predominating.	[46]

**Table 3:** The functional properties of β-glucan extracted from various sources.

Functional Properties	Source of β-Glucan	Inferences	References
Bile acid-binding capacity	Cereal	Low-Mw β-glucan bound more bile acid than did the high-Mw β-glucan (*p* < 0.05).	[29]
	*Agaricus bisporus*	Enhanced bile acid-binding was observed in low-Mw β-glucan obtained after γ-irradiation	[68]
	*Poria cocos*	Improved solubility of β-glucan after oxidation led to improved bile acid-binding capacity	[69]
	Oat	Oxidative treatment with hydrogen peroxide enhanced the sums of carbonyl and carboxyl contents of the β-glucan and also lead to an improvement in bile acid-binding	[70]
	Oat	The decrease in the molecular weight of oat β-glucan exhibited higher bile acid-binding capacity	[32]
	Oat	Acetylation of β-glucan enhanced the bile acid-binding ability	[21]
	*Poria cocos*	Carboxymethylation of β-glucan enhanced the in vitro bile acid-binding capacity	[71]
	Oat	Sulfation of β-glucan reduced the in vitro bile acid-binding capacity due to the decrease in the molecular weight of β-glucan	[20]
	Oat	Aminated-derivatized β-glucan exhibited enhanced bile acid-binding activity	[72]
	Oat	Enhanced bile acid-binding capacity was observed in oxidized β-glucan	[73]
	Oat	β-Glucan fractions with Mw 2.42 × 10^5^ and 1.61 × 10^5^ g/mol bound the greatest amounts of bile acid	[30]
	Oat	Oat slurries treated with proteinase or proteinase and α-amylase exhibit improved bile acid binding	[52]
	Oat	Bile acid binding capacities of low-Mw (157,000) and medium-Mw β-glucan fractions (277,000) tended to be greater than that of the high-Mw fraction (560,000).	[67]
Solubility	*Pleurotus tuber-regium*	Globular molecular structure of β-glucan in 0.02% NaN_3_ after microwave heating exhibit high solubility	[74]
	*Poria cocos*	Extended flexible chains of phosphorylated β-glucan exhibit enhanced solubility in 0.15 M NaCl	[75,76]
	Oat	β-Glucanase treated β-glucan exhibit semi-flexible chain to an extended random coil conformation and enhanced water solubility	[77]
	*Trichoderma* strain LE02	β-Glucanase treatment of β-glucan lead to reduced Mw and improved solubility	[50]
	*Poria cocos*	Introduction of carboxyl groups due to the oxidation of β-glucan improved its water solubility	[69]
	*Poria cocos*	Introduction of carboxymethyl groups in β-glucan improved its water solubility	[71]
	Oat	Sulfation increased numbers of small fragments of β-glucan that lead to an improvement in solubility	[20]
	Oat	Increase in the Mw of β-glucan led to a decrease in the solubility	[55]
	Oat	Oxidized β-glucan exhibit enhanced water solubility	[73]
Viscosity	Oat	Increase in the Mw of β-glucan lead to enhanced viscosity	[55]
	*Agaricus bisporus*	The decrease in the degree of polymerization of β-glucan due to γ-irradiation decrease in the viscosity	[68]
	Oat	Final viscosity of the β-glucan gel decreased with intense oxidation treatment	[70]
	Oat	The decrease in the molecular weight of β-glucan followed by enzymatic hydrolysis lead to reduced viscosity	[32]
	Oat	Acetylated β-glucan was less viscous due to lower swelling power	[21]
	Oat	The decrease in the molecular weight of β-glucan followed by sulfation lead to a decrease in the viscosity.	[20]
	Oat	Enzymatic and heat treatment reduced the peak and final viscosities of oat slurries	[52]
	Schizophyllan	The higher viscosity of schizophyllan was observed at higher Mw	[16]
Swelling power	*Agaricus bisporus*	The decrease in the swelling power of β-glucan with an increase in γ-irradiation dose due to structural disintegration	[68]
	Oat	Low-intensity oxidative treatment of β-glucan enhanced its swelling power. However, the more intense treatment led to structural disintegration and reduced swelling power	[70]
	Oat	Acetylation of β-glucan enhanced its swelling power	[21]
Fat binding capacity	*Agaricus bisporus*	An increase in γ-irradiation dose of β-glucan leads to enhanced fat binding capacity	[68]
	Oat	Oxidative treatment of β-glucan did not affect its fat binding capacity	[70]
	Oat	The decrease in the molecular weight of β-glucan exhibit higher fat binding capacity	[32]
	Oat	acetylation of β-glucan resulted in a reduction of fat binding ability	[21]
Textural properties	Oat	Lower Mw of β-glucan exhibited less impact on the batter firmness	[67]
	Oat	Gels formed by oxidized β-glucan exhibit a decrease in hardness, adhesiveness, gumminess. No significant impact was observed in gel cohesiveness	[70]
	Oat	Acetylation of β-glucan lead to reduced hardness, increased cohesiveness, springiness, gumminess, and no adhesiveness of β-glucan gels	[21]
	Oat	The mix of 50% high-Mw (1,190,500) and 50% low-Mw (31,200) β-glucan produced the hardest but the most elastic, gels.	[47]
	Oat, barley, wheat	An increase in strength of cereal β-glucan cryogels was observed with increase in its molecular size	[26]
	Oat	An increase in strength and decrease in the brittleness of oat β-glucan gels was observed with increasing in its Mw	[27]

## Abbreviations

AsFIFFF	Asymmetrical flow field-flow fractionation
DLS	Dynamic light scattering
DS	Degree of substitution
HPGPC	High-performance gel permeation chromatography
LS	Light scattering
MALS	Multi-angle light scattering
Mw	Molecular weight
NMR	Nuclear magnetic resonance
PBS	Phosphate buffer saline
RI	Refractive index
SEC	Size-exclusion chromatography
SLS	Static light scattering
HPLC	High performance liquid chromatography
MALLS	Multiangle laser light scattering method
DRI	Differential refractive index
HPSEC	High-performance size exclusion chromatography
SE-HPLC	Size-exclusion high-performance liquid chromatography
LLS	Laser light scattering
Pd	Polydispersity index
VS	Viscosity detector
RALLS	Right angle laser light scattering detector
DV	Differential viscometer
DP	Differential pressure
